# Mechanical and biological characterization of a composite annulus fibrosus repair strategy in an endplate delamination model

**DOI:** 10.1002/jsp2.1107

**Published:** 2020-07-16

**Authors:** Dmitriy Alexeev, Shangbin Cui, Sibylle Grad, Zhen Li, Stephen J. Ferguson

**Affiliations:** ^1^ ETH Zürich, Institute for Biomechanics Zürich Switzerland; ^2^ AO Research Institute Davos Davos Switzerland; ^3^ The First Affiliated Hospital, Sun Yat‐sen University Guangzhou China

**Keywords:** AF defect, AF rupture, electrospinning, FibGen, genipin, intervertebral disc, IVD, PCL, regenerative medicine, repair, spine

## Abstract

This study compares the mechanical response of the commonly used annulus fibrosus (AF) puncture injury model of the intervertebral disc (IVD) and a newly proposed AF failure at the endplate junction (delamination) on ex vivo bovine IVDs. Biocompatibility and mechanics of a newly developed repair strategy comprising of electrospun polycaprolactone (PCL) scaffold and fibrin‐genipin (FibGen) adhesive was tested on the delamination model. The study found no significant difference in the mechanical response to compressive loading between the two models. Primary goals of the repair strategy to create a tight seal on the damage area and restore mechanical properties, while showing minimal cytotoxicity, were broadly achieved. Postrepair, the IVDs showed a significant restoration of mechanical properties compared to the injured samples for the delamination model. The FibGen glue showed a limited toxicity in the AF and produced a resilient and mechanically stable seal on the damaged area.

## Graphical abstract

The present study investigates the mechanical response of the commonly used annulus fibrosus puncture injury model of the intervertebral disc and a newly proposed avulsion of the endplate junction (delamination) on ex vivo bovine intervertebral discs. Subsequently, biocompatibility and mechanics of a newly developed repair strategy comprising of electrospun polycaprolactone scaffold and fibrin‐genipin (FibGen) adhesive was tested on the delamination model.

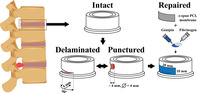

## 1 INTRODUCTION

Intervertebral disc (IVD) herniation is a very common and painful condition, where the outer layers of the IVD known as the annulus fibrosus (AF) are ruptured, which results in leakage of the inner nucleus pulposus (NP) material and consequently deterioration of the mechanical function of the organ. This damage is often sustained during intensive physical activity or due to tissue degeneration as a result of a pathology or aging. Almost 80% of the population experience lower back pain, of which approximately 40% is a result of IVD degeneration.^1^, ^2^ Herniation of the IVD can occur through the endplate in the cranial‐caudal direction, or laterally through AF failure. The injury of the AF and NP herniation are often followed by further degeneration due to immune responses, provoked inflammation and an array of circulating cytokines.^3^, ^4^, ^5^ Severe back pain symptoms arise as a result of these processes as well as NP material directly impinging on the nerve root, and vascular and nerve invasion of the injury site.^6^, ^7^, ^8^ To evaluate effective treatment methods, the injury model and the repair strategy need to be first considered individually and subsequently tested in conjunction.

To establish an ex vivo injury model which is most representative of the naturally occurring case, several types of AF ruptures that can lead to circumferential NP extrusion were considered. Two general rupture types were identified in the literature, including failure at the endplate junction and failure through rupture of the AF in central region. It has been shown that 65% of discs rupture at the endplate, where the most common mode is through rim fracture.^9^ These findings were supported by other studies documenting a frequent presence of cartilage tissue in herniated material,^10^, ^11^ as well as a weak interface at the endplate in extension loads.^12^ Failure through AF rupture in the central region is commonly investigated in the literature. Multiple studies have characterized the pathomechanism of the injury^4^, ^13^, ^14^, ^15^ as well as proposed repair strategies. The repair strategies can be divided into the strategies focused primarily on mechanical repair and sealing, largely reliant on synthetic materials, and hybrid strategies that aim not only to provide mechanical support but to also promote remodeling of the damage site through loading synthetic or tissue engineered matrices with live cells. Approaches adapted from existing strategies such as sutures^16^ have shown promising results in preventing re‐herniation in the short term. The use of hydrogels made up of collagen^17^, ^18^, ^19^, ^20^ and fibrin‐genipin (FibGen)^21^ as fillers for AF defects have shown to limit biomechanical deterioration. The fillers have also been enhanced with a retention scaffold on the IVD surface^22^, ^23^, ^24^ to further reinforce the injury site. The second class of strategies applies tissue‐engineering principles to replace the injured AF with cell‐laden scaffolds^25^, ^26^, ^27^, ^28^ or cell sheets.^29^ While the cell strategies show promising biomechanical results in small animals, as well as cell survival and proliferation, the main aim of accelerating regeneration of the injury by the addition of live cells has had limited evidence of efficacy. The more common failures at the endplate junction have not been investigated outside the clinical description. In this study, based on previously documented radiographs and MRI scans of the injuries at the endplate junction, a model most closely resembling that found clinically was established.

To repair the ruptured disc, a system that is biologically and biomechanically compatible must be chosen. Considering the inherent properties of the AF, mechanical design criteria that will allow for restoration of the original mechanical properties can be defined. The material of choice should be capable of withstanding large strains up to 40%, based on the surface strains observed in IVDs.^30^, ^31^ Additionally, a tight seal on the injured area is required to prevent nerve and soft tissue ingrowth into the injury, as well as prevent further degeneration leading to NP extrusion which can both cause severe pain.^6^, ^7^, ^32^ Furthermore, the repair construct should exhibit a modulus which is lower than that observed in the outer layers of the AF. Based on the review by Long et al,^33^ a range of mechanical properties suitable for the repair were identified.

Electrospun (ES) poly(ε‐caprolactone) (PCL) was chosen to fulfill the role of principal mechanical support. PCL is commonly used as a substrate providing mechanical support and guiding cellular activity in tissue engineering (TE) and regenerative medicine.^34^ The ES networks can be produced with wide variety of fiber diameters and morphologies depending on the production process, and thus they can closely mimic the scale of structures and fibrous morphology of the extra cellular matrix.^35^, ^36^, ^37^ The mechanical properties of these scaffolds can be tuned through alteration of spinning parameters to achieve various morphologies, fiber diameters, and alignments. Previous research has shown that PCL can achieve mechanical properties in the required range of 1 to 40 MPa^38^; furthermore, the anisotropy requirements have been shown to be fulfilled through fiber alignment.^39^ The required strain range of up to ±40% strain is also within the capabilities of the scaffolds. To attach the ES scaffolds on the surface of the IVD, FibGen hydrogel glue was chosen as it allows good fixation through chemical bonding with the IVD surface and mechanical interlock with the ES scaffolds. FibGen has been previously shown to have strong and tunable fixation for collagen rich soft tissues,^40^, ^41^ and mechanical properties in the range of native AF tissue.^42^

We hypothesized that the new endplate delamination model would provide a medically relevant damage scenario, which would be significantly different from those previously used.^4^, ^13^, ^14^, ^15^ Furthermore, we hypothesized that the repair strategy devised would allow for the restoration of the mechanical response of the damaged IVD, create a tight seal on the damage area and would be cytocompatible. To investigate these hypotheses, a bovine tail IVD model was chosen, as it is well established and provides organs of similar size to human IVDs, with comparable mechanical properties.^43^, ^44^

## 2 METHODS

### 2.1 Study design

To investigate the effect of the injury on the IVD, two types of injuries were induced (puncture and delamination). The IVDs were extracted from bovine tails and separated into three groups (intact, delaminated, and punctured) with four samples in each group. The intact group was kept undamaged as a control; the punctured group used the traditional central AF injury model, while the delaminated group applied the new damage model near the endplate. The samples were tested on a schedule described in Table 1. For the investigation of the repair strategy, samples were also separated into three groups (intact, injured, repaired) with eight samples in each group. Only the delamination model was used as the injury and is therefore referred to as just the injured group. The samples were tested on the schedule described in Table 2.

**TABLE 1:** Study design for the investigation of the effect of injury type on the mechanical response of the IVDs

Day	Test	Force range (MPa)	Frequency (Hz)	Loading time (min)	Groups
1	Compressive mechanical test *Injury*	0.02‐0.38	0.2	15	Intact
2	Compressive mechanical test	0.02‐0.38	0.2	15	Intact, punctured, delaminated
3‐6	Dynamic loading	0.02‐0.20	0.1	120	Intact, punctured, delaminated
7	Compressive mechanical test	0.02‐0.38	0.2	15	Intact, punctured, delaminated

**TABLE 2:** Study design for the investigation of the efficacy of the repair strategy

Day	Test	Force range (MPa)	Frequency (Hz)	Loading time (min)	Groups
1	Compressive mechanical test *Injury*	0.02‐0.38	0.2	15	Intact
2	Compressive mechanical test	0.02‐0.38	0.2	15	Intact, injured
3‐6	Dynamic loading	0.02‐0.20	0.1	120	Intact, injured
7	Compressive mechanical test *Repair*	0.02‐0.38	0.2	15	Intact, injured
8	Compressive mechanical test	0.02‐0.38	0.2	15	Intact, injured, repaired
9‐13	Dynamic loading	0.02‐0.20	0.1	120	Intact, injured, repaired
14	Compressive mechanical test	0.02‐0.38	0.2	15	Intact, injured, repaired

### 2.2 IVD preparation

IVDs comprising cartilaginous endplates were harvested from bovine tails (10‐12 months old), obtained from a local abattoir within 2 hours of death, and washed in phosphate buffered saline (PBS) containing 10% penicillin‐streptomycin (Gibco, Zug, Switzerland) for 10 minutes, followed by a second wash in PBS with 1% penicillin‐streptomycin. Three bovine tails were dissected for the injury model comparison, while five bovine tails were dissected for the repair strategy efficacy investigation. Six discs were collected from each tail. Average diameter was 18.36 mm ± 2.28 SD, average height was 10.90 mm ± 1.34 SD. The IVDs were assessed visually for signs of degeneration or abnormal growth. Discs were cultured overnight in Dulbecco's modified Eagle's medium (4.5 g/L glucose) with 2% fetal bovine serum (Gibco), 1% insulin transferrin selenium, and 0.2% Primocin (Invivogen, Nunnigen, Switzerland).

### 2.3 IVD injury

#### 2.3.1 Puncture model

**FIGURE 1:**
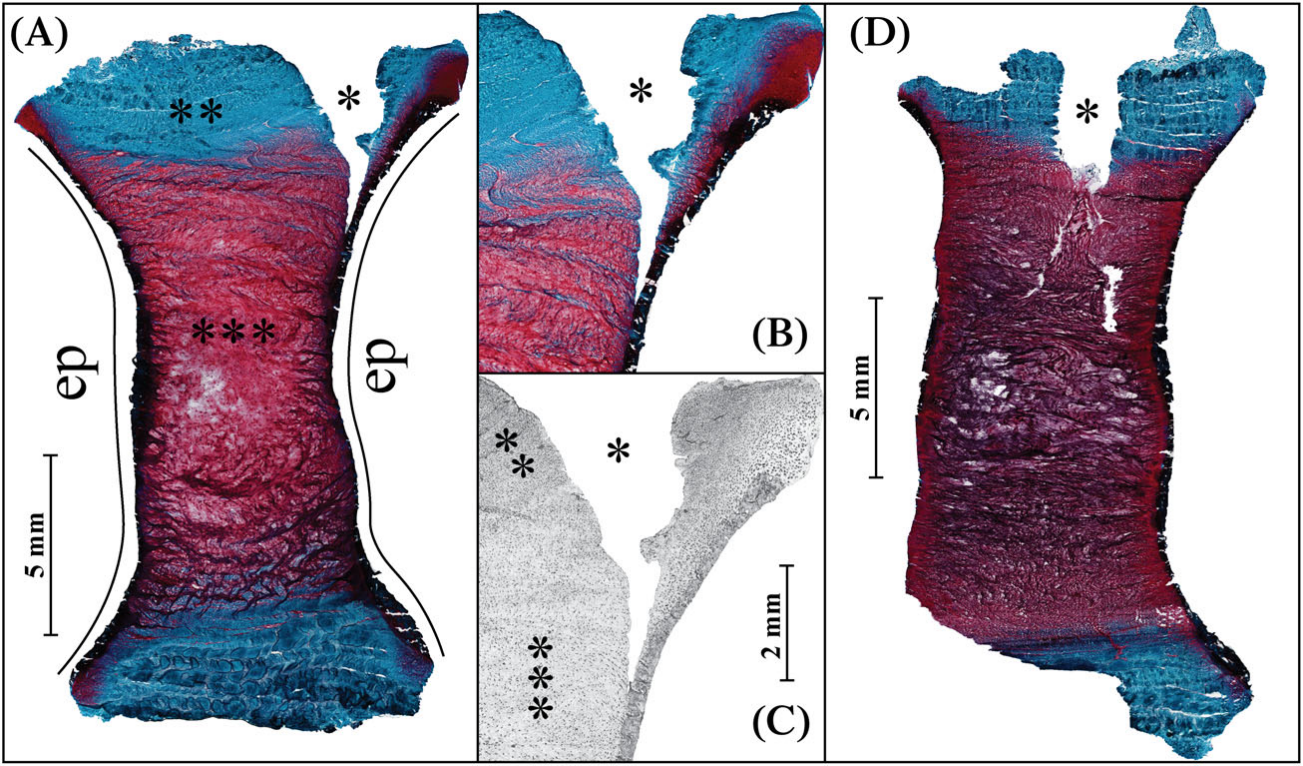
The overview of a histological section stained with Safranin‐O/Fast Green stain of the delamination injury after a total of 14 days in culture, A, here proteoglycans are stained red and collagen is stained blue, live and dead cells are stained black. A closer look at the injury site with the same stain, B, and LDH stain of the injury site with live cells stained black, C. The gradient of cell density from the highly cellularized outer AF to the NP is clearly visible in the LDH stain. An overview of the punctured disc after 7 days in culture, D. The disturbance in the NP is in present here unlike in the delamination injury. * Injury site, **AF, ***NP, EP is the endplate location. AF, annulus fibrosus; NP, nucleus pulposus; LDH, lactate dehydrogenase

The IVDs were injured using a biopsy punch (diameter 4 mm, length 7 mm, Kai Medical, Gifu, Japan) in the central region of the AF, penetrating to the NP region (Figure 1D). The methodology was adapted from Li et al.^13^

#### 2.3.2 Delamination model

**FIGURE 2:**
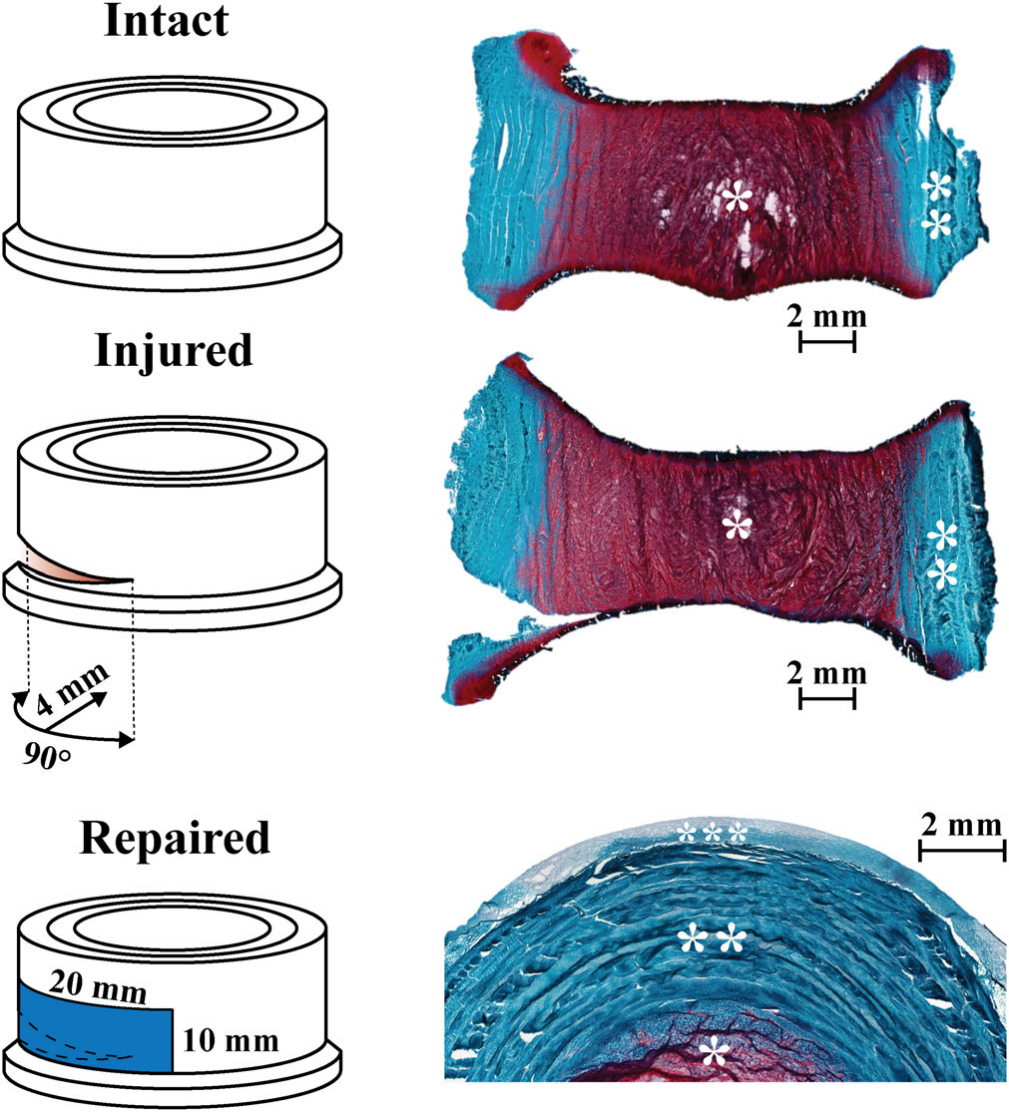
Overview of experimental design starting with intact healthy disc, the injury was induced with scalpel blade to produce an injury 4 mm deep and 90° section as close as possible to the endplate, finally the IVD was repaired using the electrospun PCL scaffold and FibGen glue. Histology images with Safranin‐O/Fast Green staining obtained after 14 days in culture and 7 days after repair was applied show the three states in which the IVDs were tested. Note the close adhesion of the repair construct to the IVD surface. * NP, ** AF, *** repair scaffold. AF, annulus fibrosus; ICD, intervertebral disc; NP, nucleus pulposus; PCL, poly(ε‐caprolactone)

The IVDs were injured using a No. 10 scalpel blade. An incision was made approximately 4 mm deep, which corresponded to the length of the curved part of the blade, as close as possible to the endplate seen on a sagittal section in Figures 1 and 2. The incision was made in a 90° section.

### 2.4 Production of the repair (ES scaffold and FibGen adhesive) and its application

#### 2.4.1 PCL scaffold electrospinning

PCL (Mn 80 000 g/mol, Sigma Aldrich Chemistry, 440 744) scaffolds were produced using 12 wt% polymer in 1:7.3 chloroform (CHCl3, Sigma Aldrich, ReagentPlus, 132950) to methanol (CH3OH, Fisher Chemicals, HPLC grade, CAS: 67‐56‐1) volume ratio solvent. IME Technologies EC‐CLI electrospinning equipment was used with a translation nozzle stage and an 8 cm diameter rotating drum collector with a 19 cm spinning distance rotating at 10 rpm. The environmental parameters were controlled at 24°C and 40% relative humidity. A needle with inner diameter of 0.8 mm and outer diameter of 1 mm was used. The source voltage was set to 24 kV and collector voltage to −2 kV. The flowrate of the polymer solution was 26 μL/min. The mats that were produced were cut into smaller pieces of 20 by 10 mm for application. The scaffolds were sterilized using a cold ethylene oxide (EO) gas procedure and finally kept under vacuum for 5 days before use to remove EO residue.

#### 2.4.2 Thrombin‐genipin and fibrinogen‐PBS hydrogel adhesive (FibGen)

Stock solution of genipin (Sigma‐Aldrich, G4796), in dimethyl sulfoxide (DMSO, Fisher chemical, D/4121/PB08) at a concentration of 50 mg/mL was prepared and stored at −20°C. Before use, 60 μL genipin‐DMSO solution were added to 140 μL of 100 U/mL solution of thrombin (from bovine plasma, Sigma‐Aldrich, T7513) in PBS solution. The mixture was then stored at +4°C. Total of 70 mg of fibrinogen (from bovine plasma, Sigma‐Aldrich, F8630) were dissolved in 350 μL of PBS + 1% Anti/Anti. The solution was kept in a water bath at +37°C and vortexed every 5 minutes to produce a homogeneous mixture. Consequently, maximum local concentrations of the FibGen hydrogel glue constituents following in situ mixing were: fibrinogen at 140 mg/mL, thrombin at 28 U/mL, genipin at 6 mg/mL, and DMSO at 11% volume.

#### 2.4.3 IVD repair procedure

**FIGURE 3:**
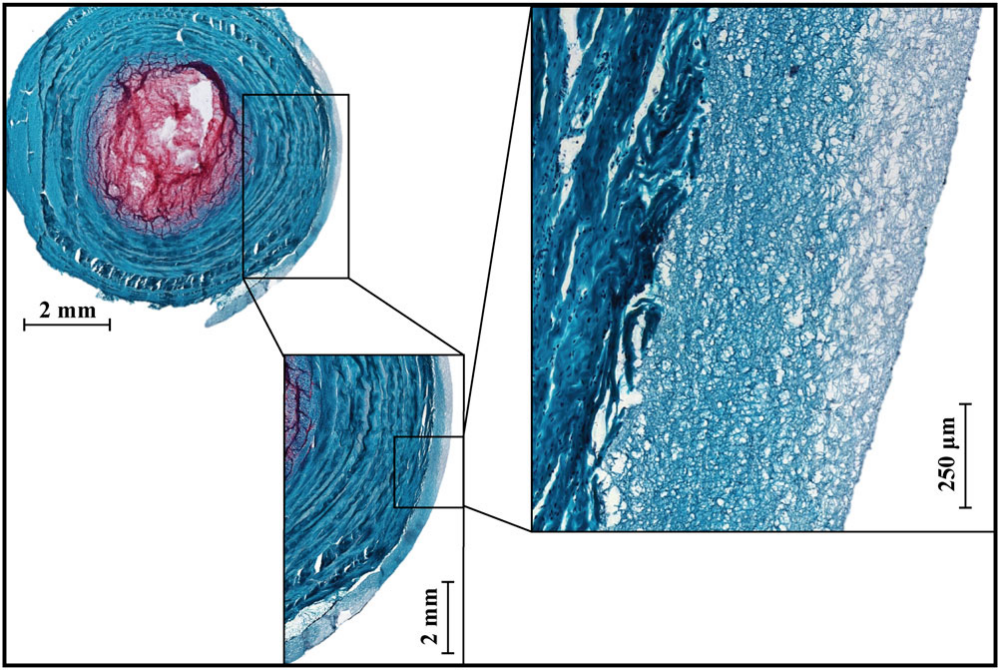
Overview *of the repair scaffold and the interface with the outer layers of annulus fibrosus (AF) on Safranin‐O/Fast Green stained horizontal section 7 days after repair procedure. Histology highlights the close adhesion and bonding of the scaffold to the outer layers of AF*

For the repair procedure, FibGen glue in two parts and the scaffold were prepared separately. Prior to application, the scaffold was soaked in thrombin‐genipin solution, followed by application of fibrinogen‐PBS on the IVD surface. The scaffold was then applied and the repaired IVDs were securely wrapped in sterilized paraffin film to create a tight seal for 20 minutes after which the repaired samples were kept in a six well plates with gauze soaked in PBS + 1% penicillin‐streptomycin (Gibco) for at least 1 hour at 37°C. The samples were then placed back in culture medium in the incubator. The resulting repair can be seen in the hystological section in Figure 3.

### 2.5 Mechanical and physical disc characterization

Two types of mechanical loading scenarios were implemented. A lower stress 2‐hour bioreactor loading protocol to simulate daily activity and a high stress 15‐minute compression mechanical testing, which was used to evaluate the mechanical performance of the IVDs. The bioreactor loading and compression testing schedule is briefly described in Table 1 for the comparison of different injury models and in Table 2 for the investigation into the repair strategy. Dimensional measurements were taken before and after each mechanical loading for both protocols. The discs were kept under free swelling conditions between all bioreactor loadings and mechanical tests. The discs were kept in custom‐made chambers during dynamic loading and mechanical compression testing, and in six well plates during free swelling.

#### 2.5.1 Dimensional measurements

The cross‐sectional measurements used to calculate stresses were taken directly after dissection using precision calipers. Cross‐sectional area was calculated assuming an oval cross‐sectional shape. Height measurements were taken daily before and after mechanical loading procedures using force limited calipers at 5 to 10 N. The height was calculated from the average of three measurements.

#### 2.5.2 Bioreactor loading

Discs were cultured in a bioreactor system with physiological loading regime. The bioreactor was maintained in an incubator at 37°C, 85% humidity, and 5% CO_2_. The dynamic loading was performed at 0.02 to 0.2 MPa and frequency of 0.1 Hz for 2 hours per day based on previously developed protocols.^13^, ^45^, ^46^, ^47^

#### 2.5.3 Compression mechanical testing

Compressive mechanical tests were done at 0.02 to 0.38 MPa and frequency of 0.2 Hz for 15 minutes with custom‐made chambers mounted on MTS Acumen 3 Test System (MTS, Eden Prairie, Minnesota). The maximum and minimum strains were recorded for each loading cycle, as well the minimum and maximum modulus which was calculated as the first 20% of strain and the last 20% of strain of the loading curve. The minimum stress in this study is referred to sometimes as the toe modulus while the maximum stress is referred to as linear modulus. The minimum strain in this study is sometimes referred to in literature as transition strain.

### 2.6 Polymerase chain reaction

AF tissue samples at the injury site and opposite to the injury site (70‐100 mg/sample) were collected from the IVDs. The samples used for gene expression analysis were snap frozen in liquid nitrogen, homogenized with 1 mL TRI reagent and 5 μL Polyacryl Carrier (both Molecular Research Center, Cincinnati, Ohio) per sample, using a Tissue‐Lyser (Retsch & Co., Haan, Germany) and centrifuged (Eppendorf, Basel, Switzerland) at 4°C for 10 minutes at 12 000*g*. RNA isolation was carried out according to the protocol from the manufacturer. Reverse transcription was performed using SuperScript VILO cDNA Synthesis Kit (Invitrogen) and 500 ng of total RNA according to the manufacturer's protocol. StepOnePlus System (Applied Biosystems) was used to conduct quantitative real‐time polymerase chain reaction (PCR). Gene expression of bovine collagen type I (Col1) and type II (Col2), aggrecan core protein (ACAN), matrix metalloproteinase 1, 3, and 13 (MMP1, MMP3, MMP13); a disintegrin and metalloproteinase with thrombospondin motifs 1 and 4 (ADMTS1, ADAMTS 4); and interleukin 6 and 8 (IL6, IL8) in disc cells was analyzed using custom designed primers and TaqMan probes (Microsynth, Switzerland) previously described in Lang et al.^47^

### 2.7 Histology

After removal of the bony endplate from both sides with a drill, the IVDs were frozen in cryoembedding compound (Sysmex, Horgen, CH). Transverse and sagittal sections (10 μm) were cut with a microtome (Microm, Germany).

#### 2.7.1 Safranin‐O/Fast Green staining

The extracellular matrix (ECM) in the injury and repair sites, as well as the native tissue was qualitatively evaluated by Safranin‐O/Fast Green staining. Sections were fixed in 70% methanol before staining. Sections were stained with 0.1% Safranin‐O and 0.02% Fast Green to reveal proteoglycan and collagen deposition, respectively, and counterstained with Weigert's hematoxylin to reveal cell distribution.

#### 2.7.2 Lactate dehydrogenase and DAPI staining

The cell viability at the injury and repair sites, as well as the native tissue was quantitatively evaluated by staining. The cryosections were stained using lactate dehydrogenase (LDH) solution as described previously.^13^ Staining was performed with LDH in 40% polypep solution (Sigma‐Aldrich, Buchs, Switzerland). DAPI staining was performed using ProLong Gold antifade reagent with DAPI (Life Technologies, Eugene, Oregon). Six IVDs per group were analyzed, where regions of 4000 by 6000 μm were imaged at the NP, AF at the injury site and AF opposite the injury site. The cells stained with DAPI were segmented by ImageJ v.1.52p software in the whole imaged region of interest; the LDH stain intensity corresponding to each nuclei was measured to identify live cells. The total number of cells was in the range of 10 000 for AF and 5000 for NP regions. The results were validated by manual count of four randomly selected small areas in each region. The cell distribution as a function of distance from the external boundary of the AF at the injury site and the intact site of each IVD were analyzed using an in house Python script.

### 2.8 Glycosaminoglycan and nitric oxide medium assays

The total glycosaminoglycan (GAG) content of the culture media, collected at each media change, was determined by the dimethylmethylene blue dye method, using bovine chondroitin sulfate as the standard,^48^ to assess the release of matrix molecules from the sample into the media. Absorbance was measured with a Victor3 PerkinElmer (Waltham, Massachusetts) 1420 multilabel counter. Levels of nitric oxide (NO) production in the conditioned medium of IVDs were determined as the concentration of its stable oxidation product, nitrite (NO^2−^), using the Griess Reagent Kit (Promega, Madison, Wisconsin).^49^ The results were calculated based on the medium volume in each well or bioreactor chamber and then divided by the volume of each IVD measured after extraction on day 0.

### 2.9 Data postprocessing and statistics

Statistical analysis was performed using GraphPad Prism 8.2 software (GraphPad Software, Inc., La Jolla, California), unless stated otherwise. The viability of the cells in LDH measurements was analyzed using independent two‐sample *t* test at each location, with Holm‐Sidak multiple comparison correction, due to the large dataset (three transverse sections from one sample and three sagittal sections from another sample, n = 6). PCR data were first preprocessed to eliminate outliers using ROUT method at Q = 1%, the normality of the dataset was then assessed and was found to be non‐parametric in most samples due to low sample number: n = 4. Kruskal‐Wallis test was then used to assess the significance of the results with multiple comparisons. The overall GAG and NO content was assessed through a Wilcoxon matched pair test, while individual time points were analyzed using an independent two‐tailed two‐sample *t* test, with intact samples as control (n = 8). The mechanical data were analyzed with the SPM1D package for Python^50^ using a two‐tailed independent two‐sample *t* test based on random field theory, with intact samples as control (n = 8). *P* < .05 was considered statistically significant (*P < .05, **P < .005, ***P < .0005).

## 3 RESULTS

### 3.1 Mechanical evaluation of the injury models and repair strategy

**FIGURE 4:**
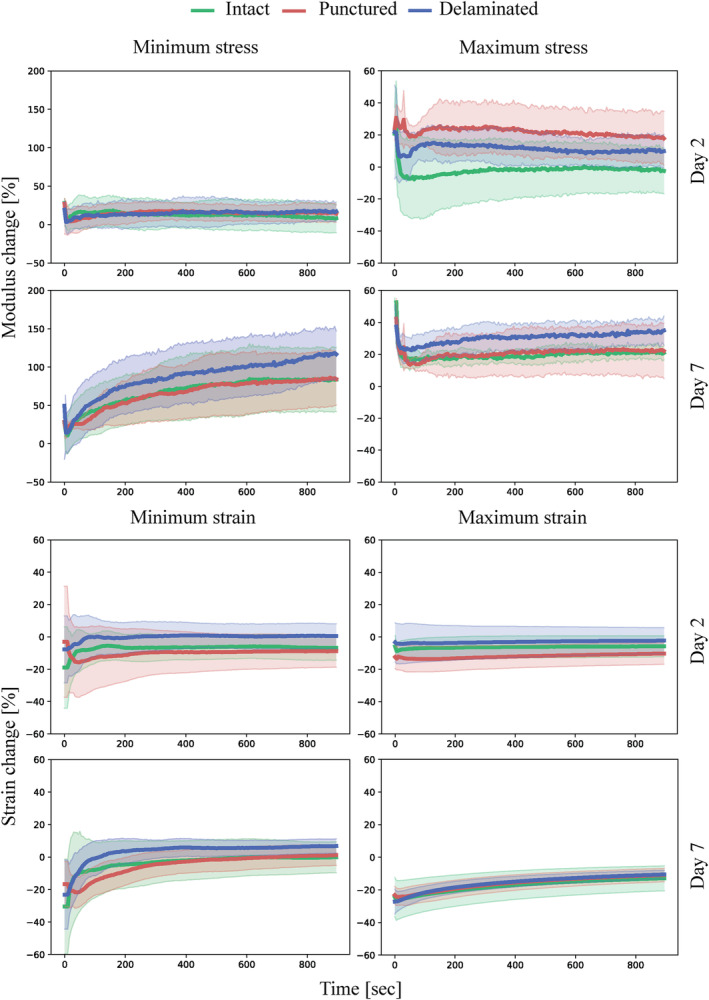
Minimum stress, maximum stress, minimum strain, and maximum strain plotted against time with shaded areas representing SD measured during cyclic (0.2 Hz) compressive loading of the samples with different injury types (punctured and delaminated) normalized to the values obtained from intact samples on day 0. n = 4

**FIGURE 5:**
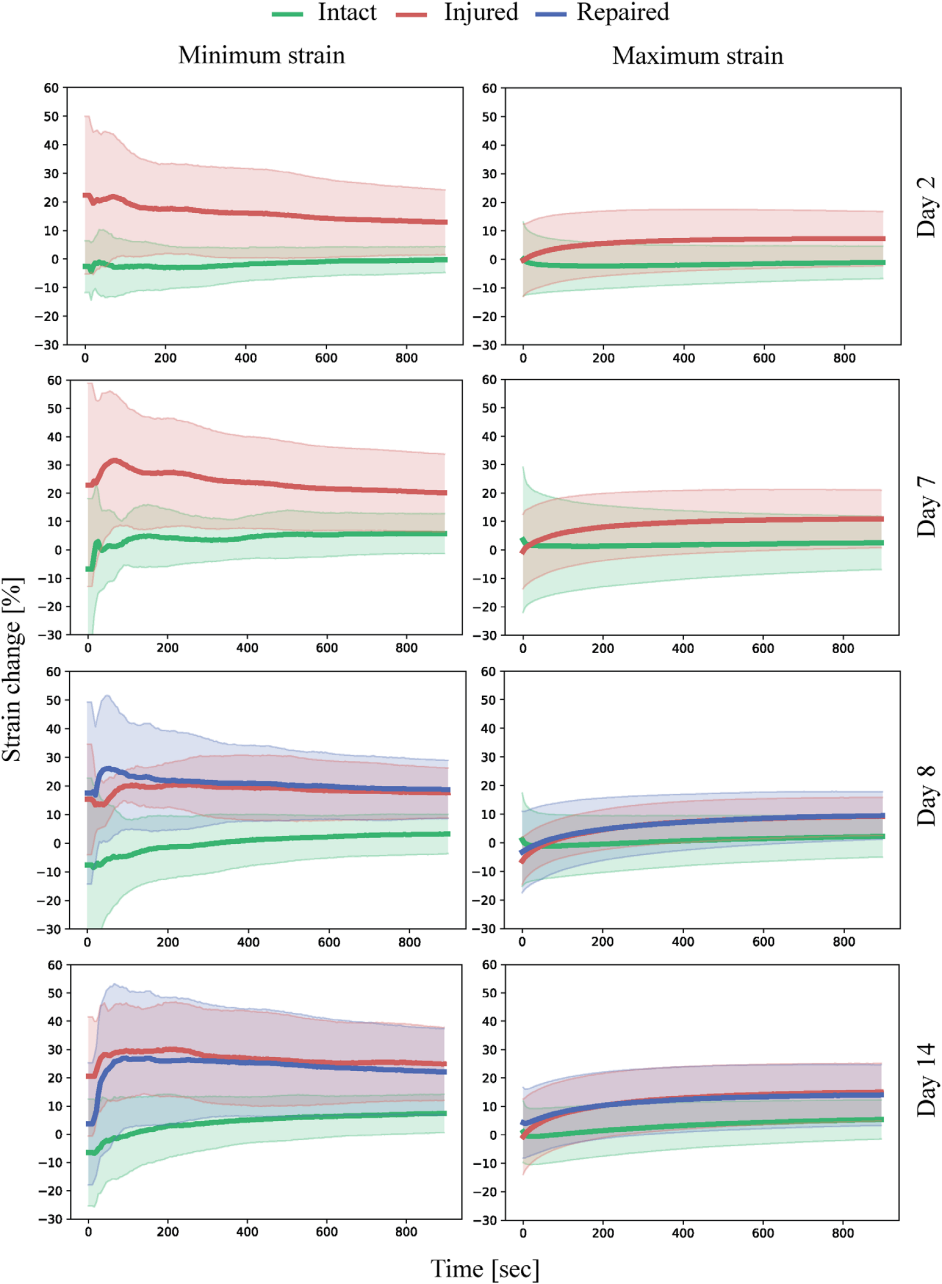
Minimum strain and maximum strain plotted against time with shaded areas representing SD measured during cyclic (0.2 Hz) compressive loading of the samples in intact, injured, and repaired states normalized to the values obtained from intact samples on day 0. n = 8

**FIGURE 6:**
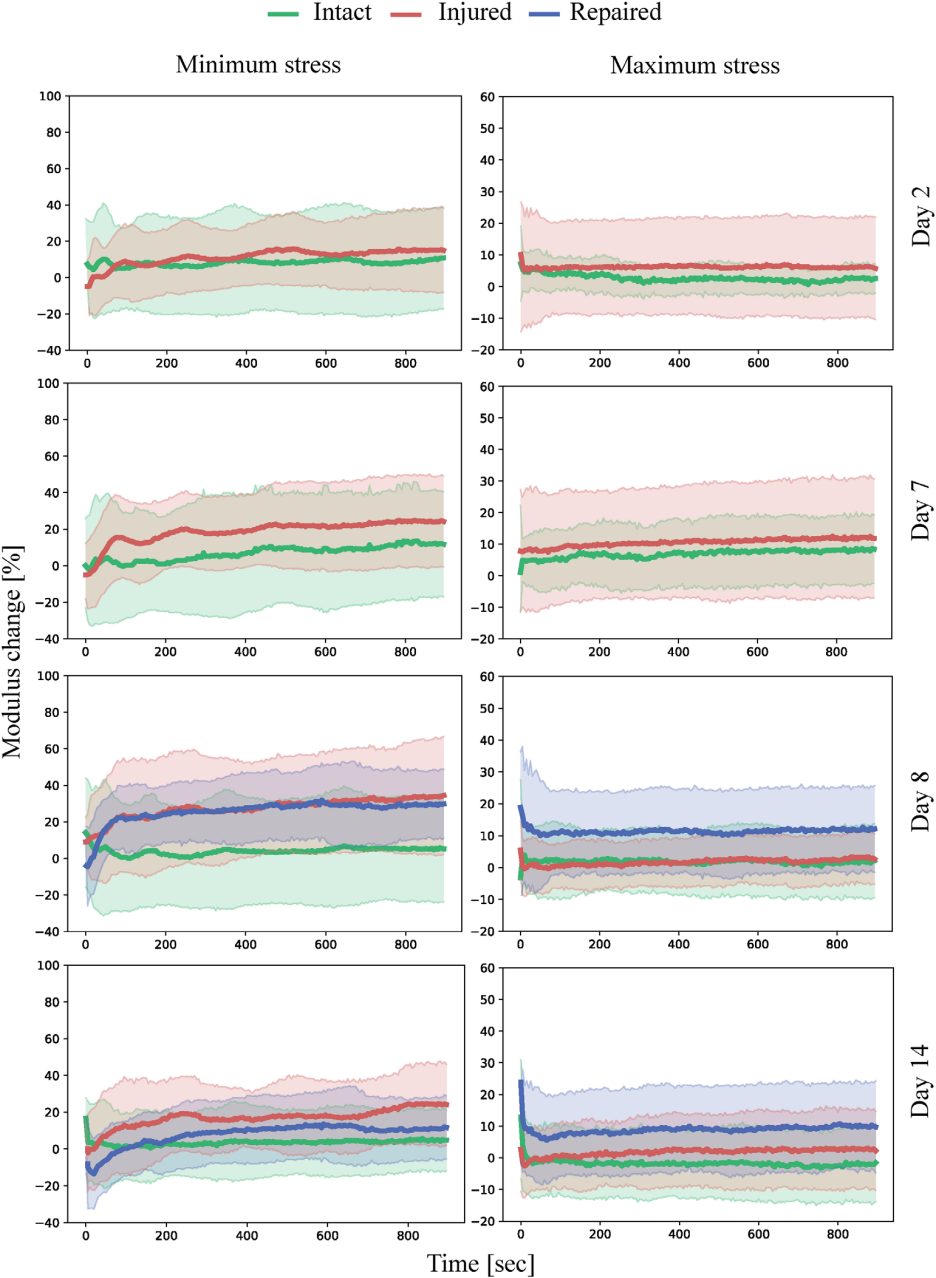
Minimum stress and maximum stress plotted against time with shaded areas representing SD measured during cyclic (0.2 Hz) compressive loading of the samples in intact, injured and repaired states normalized to the values obtained from intact samples on day 0. n = 8

Similar to previous studies,^51^ the IVDs show a hyper‐elastic and viscoelastic response where the initial loading curve exhibits low modulus in the beginning while the last part of the curve has a significantly higher modulus, as well significant hysteresis and time‐dependent behavior. For each cycle four parameters were measured (maximum strain, minimum strain, maximum modulus and minimum modulus). These parameters are plotted as function of time and sample state in Figure 4 for the comparison of the two damage models investigated and in Figures 5 and 6 for the investigation into repair strategy efficacy. The commonly reported disc height loss due to loading is not reported, as it is functionally the same as the maximum strain reached on each cycle, while also being less reliable due to manual nature of the measurement. The height measured before the samples were loaded (after recovery) did not show any significant differences between groups.

##### Mechanical comparison of the central AF puncture and endplate delamination injury models

No values were found to be statistically significant between injured, punctured, and delaminated, groups for any of the mechanical parameters measured. The visual comparison between the models is documented in a horizontal section in Figure 1.

##### Mechanical evaluation of the repair strategy on the endplate delamination injury model

The glue penetrated into the scaffold and adhered well to the AF, as evidenced by blue color of the scaffold and tight adhesion of the scaffold to the AF surface after multiple days of loading as seen in Figure 3. The adhesion of the FibGen to the AF was also successful, as all scaffolds (n = 8) stayed attached to the AF surface after 5 days of diurnal and 2 days of high stress compressive loading. No tissue ingrowth or cellularization was observed in the scaffold or the hydrogel.

**FIGURE 7:**
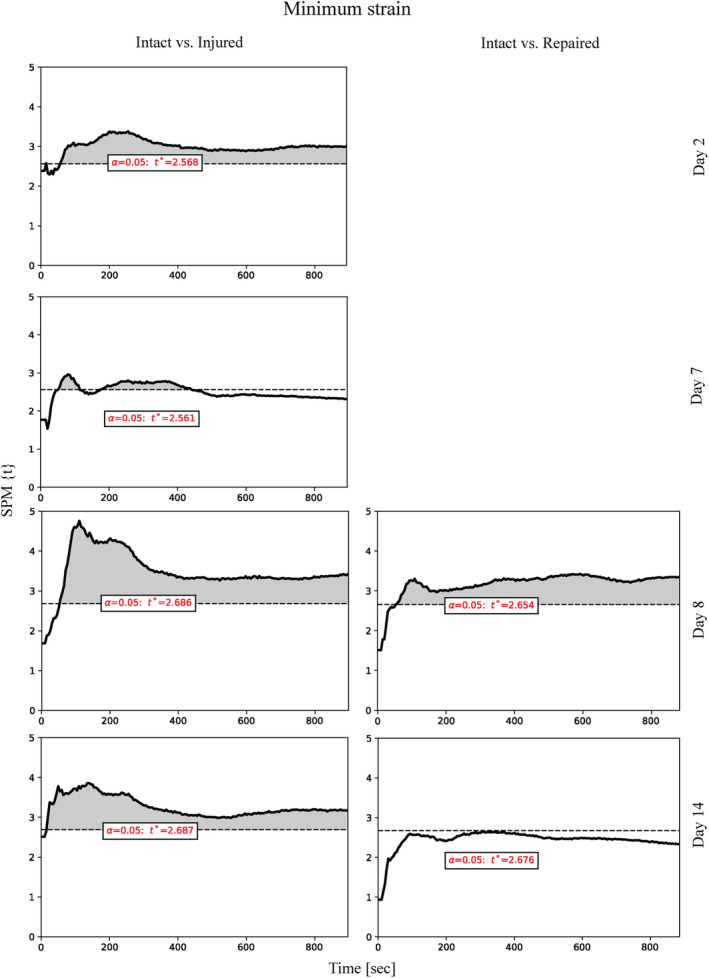
Independent two‐sample *t* test 1D analysis of the minimum strain measured during cyclic (0.2 Hz) compressive loading of the samples in intact, injured and repaired states processed using SPM1D software. The *t* values are plotted as function of time, with significance level (*P* < .05) denoted as a horizontal dotted line. n = 8

All modulus values were not statistically different for the intact, injured (delaminated) and repaired groups. The maximum strain reached during the cycle was also not statistically different for all groups. However, the minimum strain reached at every cycle, which is the strain at minimum stress of 0.02 MPa, was significantly higher for injured samples vs intact throughout the whole experiment at all time‐points as can be seen in Figure 7. The repaired sample also exhibited significantly higher minimum strain vs intact the day after the repair procedure (day 8). After 14 days, the minimum strain was not significantly different from the intact samples, which suggests restoration of mechanical properties.

#### 3.1.2 Biological effect of endplate delamination injury and repair strategy

##### Cell viability

**FIGURE 8:**
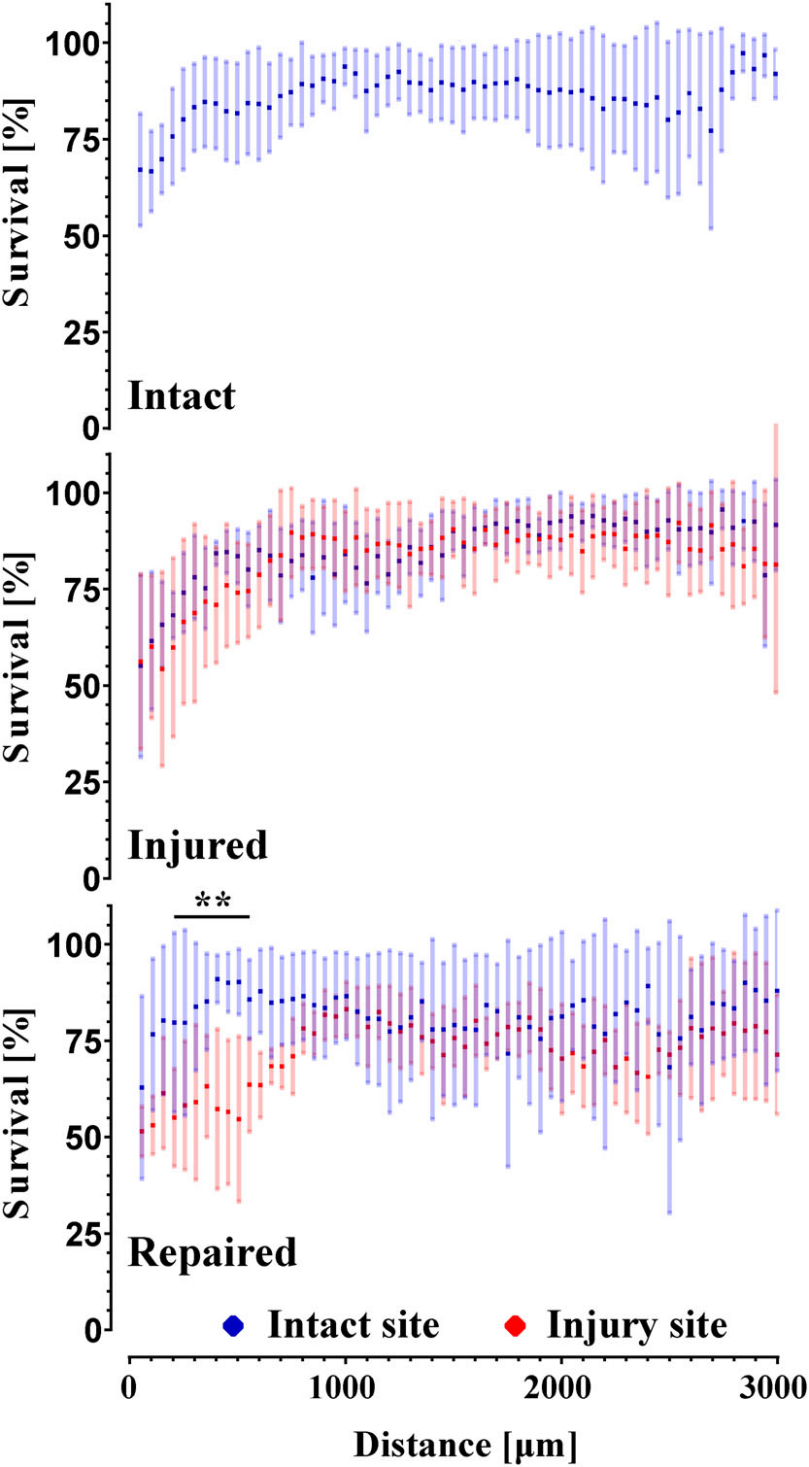
Graph of cell viability plotted as a function of distance from the surface of the intervertebral disc (IVD) (in blue from the intact site, in red from the injured or repaired site) with error bars representing SD. n = 6

Cell viability, which is affected by the ex vivo environment, injury as well as genipin toxicity, was assessed using LDH and DAPI stain on cryosections. The results presented as viability of the cells as function of distance from the edge of AF in bins of 50 μm are shown in Figure 8. It was found that the viability fraction did not differ significantly between the intact sides of all samples. The side that was damaged on the injured samples was not significantly different from the opposite intact site on the same samples; it was also not significantly different from the samples kept intact. However, the damaged side in repaired samples showed significantly lower viability from 100 to 600 μm away from the AF edge compared to the opposing undamaged side. It was also significantly different from the injured samples, as the repaired samples showed significantly lower viability from 450 to 750 μm. These findings suggest that the injury does not have a significant effect on cell viability; however, the application of the FibGen glue and the scaffold leads to significantly lower cell viability up to 600 to 750 μm from the application site. Cell viability in the NP was above 90% in all samples.

##### Gene expression

**FIGURE 9:**
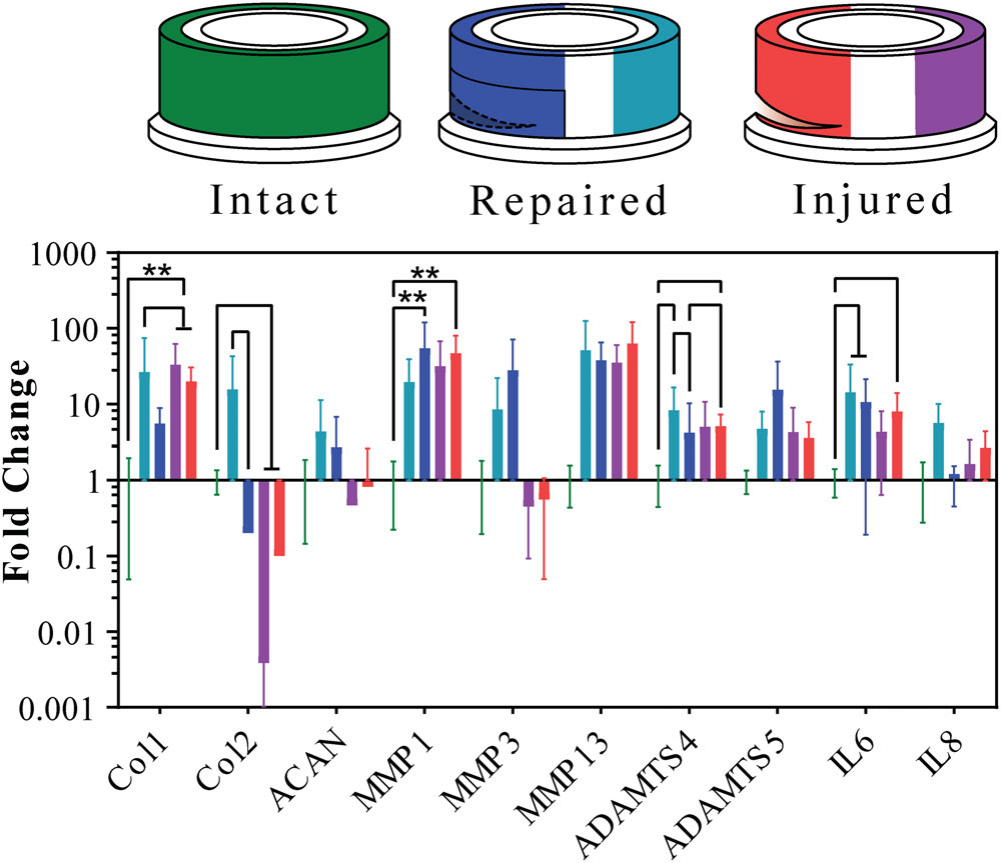
Results of the RNA quantification extracted from intact samples annulus fibrosus (AF); injured samples AF near the injury site and AF from the opposite side of the injury site; repaired samples AF near the repair site and AF from the opposite side of the repair site. Error bars showing SD, n = 4

Gene expression of anabolic genes collagen type I (Col1) and type II (Col2) and ACAN, catabolic genes matrix metalloproteinase 1, 3, and 13 (MMP1, MMP3, MMP13), as well as ADAM metallopeptidase with thrombospondin type 1 motif4 and motif5 (ADAMTS4, ADAMTS5) and inflammatory genes interleukin 6 (IL6) and 8 (IL8) was determined from AF at the injury or repair site and intact (control) side, as shown in Figure 9. Anabolic genes showed a significant upregulation of Col1 in injured samples. A significant downregulation of Col2 was observed for injured samples at all AF sites, as well as downregulation at the repair site compared to the opposite for repaired samples. The catabolic gene MMP1 showed upregulation at injury and repair sites relative to the intact control. Furthermore, ADAMTS4 gene was upregulated in repaired control and injury site relative to the intact control. The repair site was downregulated relative to the injury and repair control. Finally, the inflammatory IL6 gene was upregulated in the whole AF of repaired sample and at the injury site relative to the intact control.

##### NO and GAG medium assay

**FIGURE 10:**
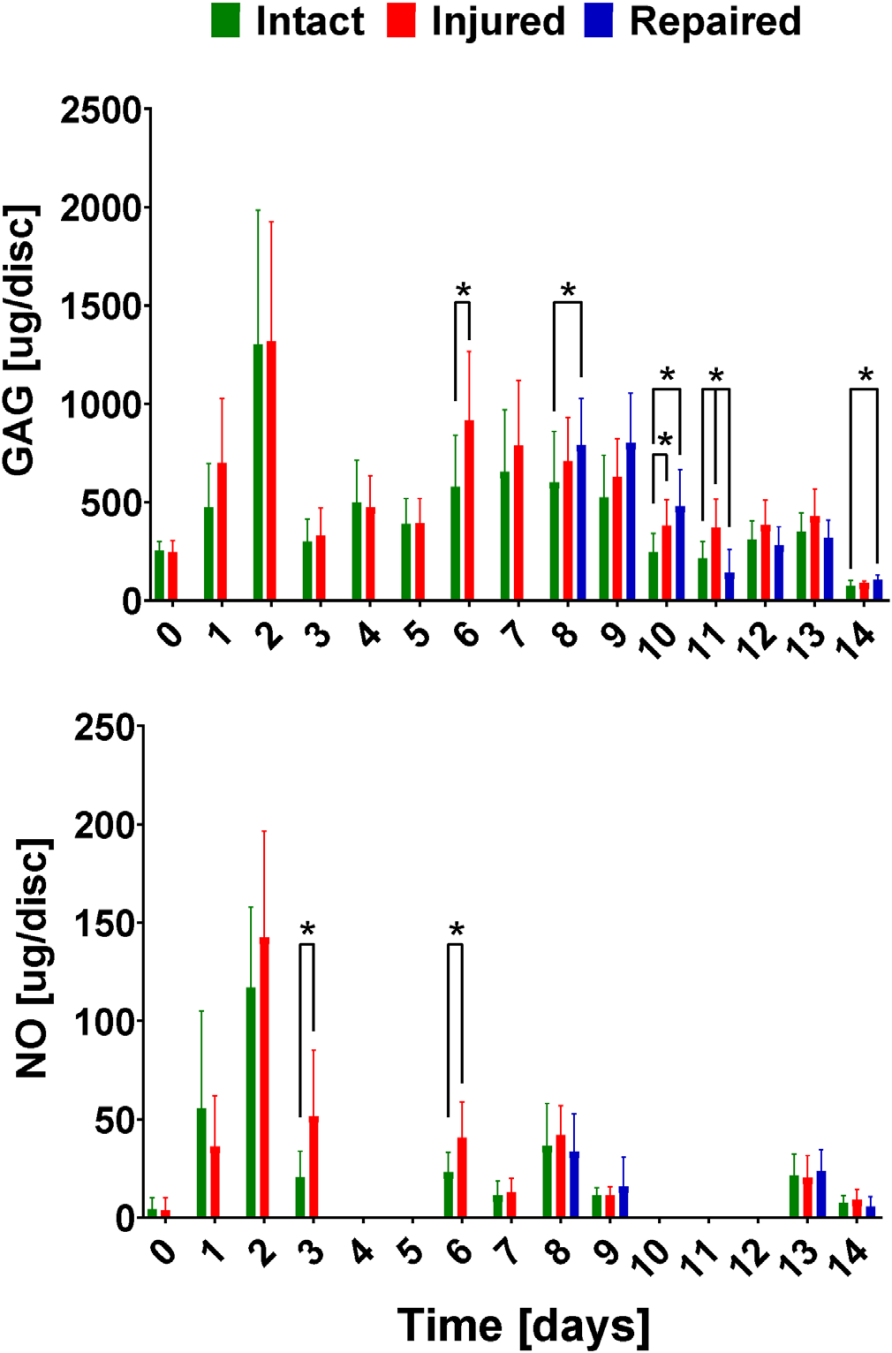
Graph of glycosaminoglycan (GAG) and nitric oxide (NO) content per disc volume plotted as a function of time. HS underlines the measurements done after the intervertebral discs (IVDs) were subjected to high stress mechanical testing. Error bars showing SD. n = 8

The GAG and NO content of the medium was measured daily from samples taken before and directly after the loading cycles. The total amounts were calculated by combining the results from directly after the loading and the amount released into the medium during the overnight incubation. The NO and GAG amounts per disc as a function of time can be found in Figure 10. The overall GAG in the medium measured in μg per mm^3^ of an IVD was significantly higher (*P* < .05) for injured samples (0.186 ± 0.103) compared to the intact (0.135 ± 0.080), there was no statistical difference between intact and repaired (0.136 ± 0.097) samples. At individual time points analyzed through *t* tests, the repaired sample initially showed higher GAG content in medium than intact samples (days 8‐10), however thereafter this observation was reversed, where the GAG content was significantly lower for repaired samples. The absolute NO values were low at all time points. The injured samples showed significantly higher levels after diurnal loading on days 3 and 6 compared to intact samples.

## 4 DISCUSSION

### 4.1 Mechanical comparison of the central AF puncture and endplate delamination injury models

The injury mechanisms of the IVD have been investigated from both the mechanical and biological perspective in a variety of scenarios. However, the relevance of the injury models to clinical observations is rarely considered. In this study we attempted to replicate the case of endplate delamination, most commonly observed in peripheral AF failure based on the clinical description by Rajasekaran et al^9^ and Veres et al.^12^ To achieve this, a circumferential scalpel incision was successfully implemented as an analog to fracture of AF at the endplate interface. When comparing Figure 1A‐C to the clinical description in literature, the morphological replication was successful, with injury at the interface of the AF and the endplate, and significant damage both in the AF and NP region.

The two injury models were expected to have different responses to the compressive loading as the geometry of the defect and its location was very different. Mechanical resistance in the IVD arises from the ability of the AF to resist tensile stresses imposed by the incompressible NP when compressive forces are applied to the spine.^52^, ^53^ Physical containment of the NP and mechanical performance of the AF are the two parameters dictating the stress strain response. The models investigated in this study are different in both those aspects. In the puncture model, the NP and AF are severely disturbed by the puncture as observed in Figure 1D and were able to immediately protrude from the injury site, while in case of the delamination injury, the NP was largely undisturbed, as seen in Figure 1A,B. This outcome is clearly the result of the geometry of the injury. This means that in the delamination injury. The changes in mechanical properties are a direct result of alterations in the tissue mechanics in the IVD, rather than a reduction in the amount tissue. This difference in geometry should also lead to a different effect on the mechanical performance of the AF. The delamination injury compromises a substantially larger number of AF fibers, compared to the puncture. In addition, it is important to note that the delamination injury scales with the IVD size unlike the puncture. In summary, the puncture injury would facilitate early NP herniation, while the delamination injury results in a much higher proportion of damage to AF. Severed tissue and NP herniation leads to an immediate initial impact on the mechanical properties. While a large delamination defect in the AF would likely not have an immediate effect, as no tissue is removed, the increased load on the NP and remaining AF would lead to increased degradation and mechanical damage to those tissues. The larger AF injury would likely also show greater differences from the intact state overall.

The two different damage models investigated in this study did not show a significant difference in mechanical response between the models or from the control that was kept intact. This is likely due to intersample variability (n = 4) in conjunction with the small impact of the injuries on compressive mechanical behavior. As ex vivo samples had to be kept alive for an extended period to evaluate the biological changes during ex vivo culture, and to provide mechanical response most similar to in vivo organs, the samples could not be embedded in a rigid mold at the vertebra. Such a procedure would limit nutrient access through the endplate and lead to degeneration. Therefore, only compressive loading was available for mechanical testing and diurnal loading protocols. More extensive differences between puncture and delamination model could have been observed through compressive loading with an angular component, as the failures at the annular‐endplate occur more often when the load is applied with increasing flexion angle which causes tensile stresses, as documented by Veres et al.^12^ However, the results suggested that the delamination model has a potential to show increased maximum modulus and increased minimum strains after 7 days in the bioreactor. These observations are likely due to the collapse of the IVD during loading and at or near endplate‐to‐endplate contact in compression, which leads to an increased modulus relative to both the intact and punctured groups. The increase in minimum strain reflects the inability of the IVD to recover height quickly after load is released, likely due to increased hydraulic permeability as noted by Grunert et al.^19^ The delamination model was therefore chosen for the second phase of the study where the repair strategy would be investigated, as it showed potential for significantly different mechanical properties.

### 4.2 Mechanical and biological evaluation of the endplate delamination injury and repair strategy

The repair strategy designed around addressing the failures at the annular‐endplate junction focused on three main goals: tight seal and adhesion to the injury site, restoration of the mechanical properties and cytocompatibility. The chosen approach comprised two components: ES PCL and FibGen hydrogel adhesive.

The overall design was based on a bioinspired composite like structure where the matrix component is a FibGen hydrogel reinforced with PCL ES scaffold. The FibGen hydrogel also acts as an adhesive component to the AF. The technique used for the application of the scaffolds relied on the interpenetration of the FibGen hydrogel and the PCL matrix, which was successfully achieved through soaking the scaffold in the thrombin‐genipin component prior to application onto the AF surface covered in fibrinogen. The adhesion process took approximately 30 minutes under ambient conditions, which meant that the membrane needed to be mechanically fixed for this period. This was achieved through fixation with paraffin film wrapped tightly around the IVD for said period. This step is similar to the pressure the surrounding tissue would exert on the repair site in an in vivo scenario. It is considered acceptable to immobilize the patient for the required 30 minutes to avoid shifting the patch. Furthermore, in vivo the IVD would be under constant pressure, which would mean that there would be minimal changes in volume and shape to facilitate good adhesion. Overall, the application process was sufficient; however, if further optimization for in vivo implementation to reduce adhesion time is required, it can be achieved through altering the FibGen formulation.^42^

The mechanical response throughout this stage of the study was focused on testing the repair strategy, as shown in Figures 5 and 6, and therefore comprised three groups: control, injured group and repaired group, which was injured and then repaired illustrated in Figure 2. The focus was placed on establishing an injury model mechanically significantly different from the intact samples, and to achieve restoration of the mechanical properties through the repair procedure. Similarly, to the comparison of the injury models, the mechanical evaluation was limited to compressive loading due to biological considerations, preventing fixation at the vertebra. However, unlike the injury model investigation, significant differences were found for the minimum strain between the intact and injured samples. This measurement corresponds to the ability of the disc to recover height under load. This ability was significantly reduced for the injured samples at all time points. On the other hand, it was restored to the intact values for the repaired samples on day 14, which corresponds to 7 days after repair. This suggests that the full crosslinking process proceeds for more than 24 hours after the application. In particular, this finding could be a result of genipin crosslinking collagen in the AF matrix, which would lead to a stiffer and more elasticity in AF. The diffusion process required for the genipin to penetrate into the AF would slow down the reaction, unlike in the FibGen hydrogel where the components are liquid and the layer is thin compared to AF, as seen in Figure 3. The stiffening of the AF on genipin exposure is further supported by Fessel et al,^54^ where increased stiffness was observed in collagen networks on application of genipin.

One limitation of comparing the biomechanics results in the current study to many other repair strategies that have been previously attempted in literature is the type of injury that is repaired. All studies that are cited here use various types of puncture injuries in the central AF. For small animal models, a needle puncture is used or AF tissue removal for larger defects, for large animal models a biopsy punch is used or once again AF tissue is removed directly. As discussed in the comparison of the injury models, the commonly studied puncture injury likely has a different mechanical effect than the delamination injury. With that in mind, the minimum strain in this study can be considered analogous to the height loss of the IVDs postloading, which is commonly reported in literature. Many studies have recorded disc height loss after AF injury, and disc height is considered a benchmark for both degeneration and success of the repair strategy clinically.^55^ The results in this study are in agreement with multiple studies that have shown successful repair of the injuries to the central AF in vivo.^18^, ^23^, ^27^, ^28^, ^29^ Both in terms of significant height loss in injured IVDs and disc height restoration and improvement due to repair. These studies were done on small animal models, where the size of the defect is smaller and biomechanical requirements on the repair construct are much lower than in large animals. Pennicooke et al^20^ and Long et al^24^ who have attempted repair strategies on large animal models in vivo did not record a significant improvement in disc height on repair.

The overall IVD modulus measurements in this study, however, did not show significant differences between groups. This finding is in agreement with study by Likhitpanichkul et al,^21^ where no significant change in stiffness was also observed on a whole organ model of an IVD, although the injury and repair had different geometry than the present study.

The impact the injury and repair have on the cell viability was measured through LDH‐based live/ dead staining. The injury was found to have no effect on cell viability after 14 days. However, there was a significant decrease in cell viability on all exposed surfaces of the organ relative to the bulk in all samples, which could mask any effect the injury would have on the tissue directly adjacent to the surface. The decrease in viability on the IVD surface is consistent with findings of Li et al.^13^ Genipin has a significantly negative effect on cell survival up to 750 μm from the application site. This finding is in agreement with many studies performed ex vivo,^22^, ^54^, ^56^ where genipin of similar concentration to the present study in combination or without DMSO showed a strong cytotoxic effect. The cytotoxicity findings were supported by studies done in vitro.^40^, ^42^ On the other hand, it is in contradiction with some previously published work which showed no effect on cell viability in ex vivo organ cultures^21^ and in vivo,^57^ although the exact recipes for the FibGen hydrogel vary between the studies, as well as the geometry of the injury and the repair. The reasons for this disagreement most likely come from large variety of formulations and culture conditions used, as well as insufficient documentation of the exact absolute amount of both DMSO and genipin each cell is exposed to.

Although the toxic effect of genipin is never desirable, a compromise should be considered to provide a good mechanical support and seal on the damaged area in the absence of alternative adhesives with compatible mechanical properties. The specific mechanism by which genipin affects cells is not clear in this case, whether this is due to cytotoxicity or the environmental changes that genipin induces through crosslinking the collagen matrix. The first case is in contradiction with findings of Likhitpanichkul et al,^21^ where cell infiltration and no cytotoxicity was shown after 6 days in a bioreactor. If, however, free genipin molecules are an issue, in vivo environment would provide more continuous motion and fluid exchange, which could potentially further reduce the effect of the genipin. This assumption is supported by in vivo study by Long et al which showed no long‐term cytotoxic or inflammatory effect from genipin in ovine IVDs.^57^ In the latter more likely case, the reinforcing effect would stand in direct contradiction with cell survival. To reduce the exposure of the bulk of AF to the genipin, FibGen could be allowed to gel briefly on the scaffold prior to application, hence reducing the amount of freely available genipin. The adhesive FibGen hydrogel can be substituted for a more biologically friendly material. Riboflavin crosslinked collagen hydrogels avoid the use of potentially toxic genipin as crosslinker and have been shown to promote cell infiltration and effectively seal AF defects in small animal models in vivo and restore some aspects of biomechanics.^19^, ^27^, ^28^ Whether this approach can translate to large animal models is questionable as the biomechanical environment is far more sever. Further doubt is cast in work by Pennicooke et al, where the efficacy of such hydrogels in ovine in vivo model was investigated and found that while histologically the quality of the NP was improve, the biomechanics and disc height loss was not significantly different from the injured IVDs.^20^ This is likely due to insufficient stiffness of the collagen hydrogels required for large animal models. FibGen shows compressive elastic modulus on the scale of 100 to 200 kPa,^58^ while high‐density collagen gels are an order of magnitude softer at approximately 3 kPa.^59^ In addition, the need for UV light for crosslinking would not allow for the use of opaque ES membranes for resilience. Cyanoacrylate glues are widely used clinically, however the brittle nature of these glues coupled with potential toxicity concerns^60^ would not give them an advantage over the use of FibGen. As such, the compromise of using potentially toxic genipin as crosslinker has to be considered. An improved procedure minimizing tissue exposure to genipin and DMSO can provide the best possible outcome.

No cell infiltration into the scaffold was observed after 7 days in culture. The location of the repair as well as its geometry are likely the reasons for no observed infiltration in this study. In Figure 8, it can be seen that there is proportionally more live cells in inner regions of AF as opposed to the surface. Therefore, the application of the scaffold on the very outer surface of the AF in this study limits the cells access to the scaffold. Furthermore, FibGen is embedded in the ES fiber network with the porosity in the range of 1 μm, which is at the lower limit of porosity large enough to allow for cell infiltration. Even in the presence of cells, cell infiltration would be dependent on degradation and removal of FibGen to access the pores for proliferation. The migration of cells to the surface of the AF and degradation of FibGen would take longer than 7 days assessed in this study. A study by Likhitpanichkul et al^21^ observed cell infiltration into FibGen hydrogel after 6 days in a bioreactor on a large animal model with a central AF defect. However, unlike in this study, FibGen hydrogel as a large mass was implanted into a defect in the central AF bordering NP. In addition, the hydrogel did not contain a secondary network. Access to the large cell population present in the inner AF, was likely the main cause for the observed difference in cell proliferation.

As discussed, genipin can have a negative effect on cell survival overall. To improve the promote AF injury healing and remodeling live cells can be added to the FibGen hydrogel. Several studies have shown promising results with ECM generation and proliferation of cells embedded in both FibGen and crosslinked collagen hydrogels^25^, ^26^, ^27^, ^28^ applied to AF defects. The repair approach has shown a restoration of mechanical properties in this study, to further enhance the efficacy of the repair, especially in the long term, adding live cells to the repair construct can be considered. There are conflicting evidence regarding cell survival in genipin rich environment, as discussed previously. The proposed repair strategy would need to be tuned to ensure good cell survival, proliferation and functioning by potentially increasing the pore size of the ES membrane and adjusting FibGen composition and application process. Likhitpanichkul et al have shown cell infiltration into FibGen hydrogel when applied to AF defect^21^; it is therefore likely that such an approach is possible. On the other hand, it is unclear whether cell loading would be beneficial in this injury model as the repair is applied to the outside layers of AF only, and there is no tissue that is removed or herniated. Once the injury is tightly sealed and the IVD is once again loaded, the separated AF layers are put in contact again, therefore no hydrogel filler is necessary, and repair can proceed through the existing cells in the AF.

Cellular response to the injury and repair that was assessed through mRNA expression at the AF injury site and in the AF opposite the injury site; in addition, GAG and NO levels in the medium were measured. The upregulation of Col1 in injured samples is consistent with trend observed by Frauchiger et al, although the results there were not significant.^22^ The downregulation of Col2 in injured samples is also consistent Pirvu et al,^26^ it was also documented as part of degenerative process without inducing an injury by Lang et al.^47^ Very large and significant upregulation of MMP1, ADAMTS4 and a trend of upregulation of MMP13 in the AF of the repaired and injured samples at the injury site was not consistent with previous studies, where downregulation of catabolic genes was observed with diurnal loading.^22^, ^26^ As most markers (anabolic and catabolic) were upregulated in the AF, this may be related to a response to the injury of repair and remodeling of ECM in the AF.

The GAG content measured in the medium showed a higher value in injured samples compared to intact ones at most time points. This is likely due to increased surface area and ECM breakdown/ remodeling in the injury group. The repaired samples showed a significantly lower amount of GAG content in the medium than the injured samples 3 days after repair. This further supports the idea that the full reaction and sealing of the defect happens slowly after the initial setting and adhesion of the hydrogel. The levels of NO found in the media, which were higher for injured samples than other groups are in agreement with previous work done by Likhitpanichkul et al.^21^

### 4.3 Limitations of the study

The chosen ex vivo bovine IVD from the tail model used in this study has limitations in terms of transfer to in vivo human clinical applications. Further in vivo experiments on large animal model are required to optimize the application procedure and confirm its viability. The mechanical properties and size are similar between the human and bovine IVDs.^44^ However, biologically, the tissue compositions as well as cell types vary significantly between the two models.^61^ In particular, discs extracted from the tails of calves in this study are likely most representative of IVDs from healthy young humans.

The delamination model does not address the NP herniation and potential further tissue degeneration of the AF and NP. This limitation arises due to the nature of the injury being surgical rather than induced through degeneration of loading to failure. Therefore, it is representative of early stages of AF tare at the endplate. The repair in the presented form would need to be applied soon after the injury occurs and would likely not be suitable for severely degenerated discs, as it requires relatively healthy AF of adhere to for mechanical stability. In the future, it would be possible to explore injuries induced through flexural tensile loading of the disc, which is most likely to lead to delamination of the AF at the endplate.^12^, ^62^, ^63^ To assess the degenerative effects of the injury a much longer ex vivo study is required likely involving alterations in the IVD culture to induce degenerative changes mechanically or chemically.^47^

## 5 CONCLUSION

AF rupture is a common condition that can lead to severe pain and reduction in mobility. A new injury model similar to failure of the AF at the endplate junction was investigated and was found to be mechanically not significantly different from previously described rupture of the AF in compressive loading. A repair strategy utilizing ES PCL scaffold and FibGen adhesive was investigated through a long‐term ex vivo biomechanical testing protocol. The repair strategy showed a promising restoration of mechanical properties to the levels found in intact IVDs, while FibGen adhesive showed limited cytotoxicity in the AF. In addition, the adhesion of the scaffold to the injury site had a good seal and remained intact throughout the procedure. Individual components of the repair were not tested separately; hence, their specific contributions to the restoration are not reported. Furthermore, the degradation rates of the components may be significantly different and require further tuning. The application technique used in this study may not be suitable for in vivo procedure and may require further optimization. As such, to conclusively define the efficacy and biocompatibility of the proposed approach an in vivo study would be required in the future.

## CONFLICT OF INTEREST

The authors declare no conflicts of interest.

## AUTHOR CONTRIBUTIONS

Dmitriy Alexeev substantially contributed to the conception of the work, acquisition and analysis of the data, drafting and revising of the manuscript. Shangbin Cui substantially contributed to the acquisition and analysis of the data, as well as revising of the manuscript. Sibylle Grad substantially contributed to the conception of the work, data interpretation, and revising of the manuscript. Zhen Li substantially contributed to the conception of the work, acquisition and analysis of the data, and revising of the manuscript. Stephen J. Ferguson substantially contributed to the conception of the work, data interpretation, and revising of the manuscript.
